# The metonymic mechanism of English translation of Chinese intangible cultural heritage terms from the perspective of cognitive psychology

**DOI:** 10.3389/fpsyg.2022.957485

**Published:** 2022-07-22

**Authors:** Liping Jiang, Ghayth K. Sh. Al-Shaibani, Fenglin Yang, Mengmeng Cheng, Minghuan Huang

**Affiliations:** ^1^School of Foreign Languages and International Business, Guangdong Mechanical and Electrical Polytechnic, Guangzhou, China; ^2^Department of Education, Faculty of Social Sciences and Liberal Arts, UCSI University, Kuala Lumpur, Malaysia

**Keywords:** cognitive psychology, intangible cultural heritage terminology, Chinese-English translation, metonymic mechanism, metonymic process

## Abstract

The translation of Intangible Cultural Heritage (ICH) terms is an important practical aspect of cross-lingual expressions related to ICH knowledge. Chinese ICH terms are heavily loaded with specific historical and cultural knowledge and regional characteristics. Based on cognitive psychology, this paper analyzes the metonymic mechanism of traditional translation techniques such as substitution in the English translation of Chinese ICH terms. The English translation of Chinese ICH nominal terms can be realized based on the metonymic mechanism of replacing either a superordinate with a subordinate of the linguistic structure or a subordinate of the linguistic structure with a subordinate of the linguistic structure in the pair language, English-Chinese. The English translation of Chinese ICH verbal terms can regard the whole verbal action as an event process and can highlight one aspect of the process through a metonymic mechanism as well. This paper holds that metonymy is an important psychological motivation and cognitive mechanism for translation skills such as substitution as it brings the relationship between the corresponding items of the target language and the source language into a unified interpretation framework. Hence, this may add some significance to the research of translation theory and translation practice. This also contributes to the UN Sustainable Development Goal number 17 which seeks global parternship for sustainable development.

## Introduction

Intangible Cultural Heritage (henceforth ICH) terms are special and occur in the form of words or phrases, and they possess specific semantic characteristics and cultural connotations. The English translation of ICH terms plays an important role in inheriting civilization, expanding publicity, introducing foreign capital, and improving cultural influence. However, first, the ICH terms are a heavy carrier of specific historical knowledge, typical regional characteristics, and profound cultural connotations (Chen, 2017). Second, the asymmetry of Chinese and western languages and cultures and the cultural metaphor in ICH terms limit and restrict information dissemination of the Chinese translated texts into English. For example, the Chinese ICH term “卖身节” cannot be translated into “A festival of selling one’s body” literally; however, it should be better translated into “Selling-Oneself-for-Employment” Festival based on the fact that the term “卖身节” has nothing to do with the literal name of “Selling one’s body” because it is meant for seeking a job in old times of China. The existing translation studies generally have detailed explanations of translation skills such as substitution, addition, and subtraction; nevertheless, they have rarely discussed the cognitive psychological mechanism behind these skills. Thus, translators seldom consider the motivation and cognitive mechanism of the methods and skills used that are not conducive to translators’ flexible and effective use of these methods and skills. With the rise of cognitive science, metonymy is regarded as a conceptual mechanism and an important mechanism for human beings to understand the world, express ideas, and organize meanings. Cognitive psychology examines how people perceive, learn, remember, and think (Zeng, 2007), as well as how people learn, store, and use knowledge, and that its research scope includes attention, perception, memory, and thinking (Yu, 2021). Cognitive linguistics defines metonymy as a cognitive psychological process; that is, a conceptual entity creates a psychological channel for another conceptual entity in the same cognitive domain or idealized cognitive model (Radden and Kövecses, 1999, p. 35). Some scholars even pointed out that language is metonymic in essence. For example, metonymy is an indispensable operating mechanism and a means of translation (Pantherp and Radden, 1999). Some scholars even believe that translation is a metonymic psychological process (Tymoczkom, 1999). The Chinese scholar Wang pointed out that metonymy is one of the mainstream theories used to explain the translation process at present (Wang, 2019). Denroche put forward the metonymic translation theory of translation, which holds that the relationship between the target language and the source language is metonymic (Denroche, 2015), and the translation process is to deal with the corresponding relationship between the two at all levels through the metonymic mechanism (Li and Qi, 2012).

Bell’s model (Bell, 1991) is based on psycholinguistics and cognitive science and reflects the cognitive orientation of translation studies (i.e., the cognitive psychological process of bilingual transformation). When the translator accepts the ICH terms of the translation task, the source language input begins. At this stage, the translator reads the literal meaning of the sentence, which includes ICH terms, and then seeks its non-literal meaning. With the input stimulation, the translator begins to search the memory of words in the brain and make choices. After the pattern recognition, the translator begins to seek relevant ICH knowledge in the long-term memory for coding, which is a language creation process. After that, the translator begins to edit and correct his/her own translation and modify the inaccurate words, which is the final stage in the process of translation production. Based on cognitive psychology, which emphasizes the process rather than the result of translation, it has some enlightenment for such translators to understand the metonymic mechanism of ICH terms in English translation.

These views not only directly enhance the overall view of metonymy on human cognitive psychological ability and communicative competence, but also enhance the boosting effect of metonymic psychological thinking on the translation process. In view of this, this paper attempts to employ cognitive psychology to investigate the metonymic mechanism and cognitive rules behind translation methods and skills of ICH terms. Theoretically, this study can provide a new perspective for the study of the translation of ICH terms from the perspective of cognitive psychology and can enrich and develop the research scope of translation of ICH; in practice, it can help translators in the translation practice of culture-loaded ICH terms, provide teaching methodology for the teachers to carry out the translational course and help in the translation for the students to understand the psychological process of ICH terminology translation. It is also hoped that this contribution can fill the gap in the literature on this topic.

## Literature review

### Metonymic translation process and its applications

The conceptual study of metonymy originated from the work of Lakoff and Johnson (1980). The Chinese scholar Wang (2017) pointed out that metonymy is one of the mainstream theories that can be used to explain the translation process. The translation is a metonymic category switching activity, and metonymy is embodied in the process of translating categories (Yu, 2020). In category switching, the translator first understands and deconstructs the source language category and then finds the corresponding category in the target language for construction and expression. This process reflects the metonymic operation of “one category replacing another.” The essence of metonymy is referential, and its mechanism is X replaces Y, such as replacing a subordinate with a superordinate, replacing a superordinate with a subordinate, replacing a subordinate with a subordinate, replacing a place with an event, replacing a place for organization, and so on (Han and Wang, 2020). Tymoczkom put forward the metonymy of translation which holds that translation establishes a connection between the source language and the target language to obtain a proximity relationship (Tymczkom, 2002). The phenomenon of “a subordinate replaces a superordinate” or “a superordinate replaces a superordinate” is inevitable. Denroche put forward the metonymic theory of translation for the first time, holding that the relationship between the target language and the source language is metonymic, and translation metonymy is a bilingual information processing and transformation mechanism that exists in the process of translation (Denroche, 2015). In the process of translation, there is a metonymic relationship between the corresponding items of the target language and the source language (Lu et al., 2014). The Chinese scholar Lu called the translation of metonymy and the semantic metonymic relationship between the corresponding items of the target language and the source language in the process of translation “translation of metonymy” and “translating metonymy,” respectively (Lu, 2011). According to the perspective of translation metonymy, metonymy, as a universal cognitive psychological process, can be found everywhere in translation activities because the source text contains a series of potential meanings involved in the translation process, and the translator only selects the meaning he/she wants to represent. This paper holds that all cross-language operations based on the transformation of proximity relations, such as a superordinate and a subordinate, a subordinate and a subordinate, cause and effect, a place of origin, and a product are associated with or related to the metonymic psychological process. Some examples are shown in Table 1.

**TABLE 1:** Examples of metonymic process in translation.

Source language	Target language	Metonymic type
(1) 中国 (a country name)	China (not a country but chinaware)	Product (subordinate) replaces origin (superordinate)
(2) England won the game	英国 (Great Britain) 赢得了这场比赛。	A superordinate replaces a subordinate
(3) 她的男人 (man) 去上海打工了。	Her husband went working in Shanghai.	A subordinate replaces a superordinate
(4) 端午节 (Traditional festival)	Dragon Boat Festival	A subordinate replaces a subordinate

As shown in Table 1, when the Chinese text of 中国 is translated into English *China*, the source language 中国 and the target language *China* reflect the cross-language metonymic relationship between the place of origin and the product or the subordinate and the superordinate. In the second example, the English word *England* is translated into 英国 for the reason that England is one of the important parts of Great Britain, and the relationship between Britain and England is a relationship of superordinate category and subordinate category. The transformation of England into *Great Britain* involves the metonymic thinking of the superordinate replacing the subordinate. Replacing the lower category *England* with the upper category *Great Britain* will not confuse people and avoid offending sensitive topics. In the third example, the Chinese word 男人, which means male in gender, is translated into the English word “husband.” In Chinese, the word 人 in China has two meanings: one is a male human being and the other is a husband. In other words, *man* is the superordinate category and “husband” is the subordinate category. The relationship between the former and the latter constitutes a superordinate and a subordinate. Using the subordinate category *husband* to refer to the superordinate category *man* is a translation metonymy phenomenon of substituting a subordinate for a superordinate. Pertaining to 端午节, it is translated into *Dragon Boat Festival*. In ancient Chinese, the character 端 has the meaning of beginning, and the character 午 is harmonic with the Chinese *five*. Therefore, the fifth day of May every year is called the *Dragon Boat Festival*. In addition, people hold dragon boat races at every Dragon Boat Festival. “Dragon Boat” and 端午 both feature the attribute characteristics of the festival, and both belong to a part of the festival. The transformation of “Dragon Boat” with 端午 leads to the metonymic relationship of replacing a part with a part between the source language and the target language.

Denroche believed that the relationship between the target language and the source language is metonymic (Denroche, 2015). This relationship covers the whole language category, from a single word to the whole text and genre. The process of translation is to deal with the corresponding relationship between the two at all levels through the metonymic mechanism, including the translation of terms. Some ICH terms are heavily loaded with historical and cultural elements. Scholars generally agree that translation metonymy is caused by language and cultural differences. It is undoubtedly a beneficial attempt to apply translation metonymy theory to the translation of ICH terms.

### Intangible cultural heritage terminology and translation issues

Terminology plays an important role in improving cognitive ability, thus imparting professional knowledge and strengthening discipline construction. Terms are linguistic references to concepts in the field of professional knowledge (Wei, 2010). Terminology refers to special terms used in a discipline (Wei and Zhao, 2012); it can be seen that “specialty,” “concept,” and “reference/term/symbol” are the keywords used in term definitions. The word *term* has two etymologies: Latin etymology *terminus* and Greek etymology *terma*. The original meaning of both refers to the boundary, limit, and endpoint of the site or place, and its extended meaning is *boundary*, that is, the boundary of specialty or the limitation of a concept. Therefore, it can be briefly said that terminology is a conceptual reference in the professional field. Generally speaking, terms are unified appellations of specific things in a specific field. A term is a special word or phrase in a specific subject field that appears in the form of words or phrases and has specific semantic characteristics and cultural connotations. Terminology is the starting point of academic research and theoretical construction. Whether standardized or not, it directly affects discipline construction and international exchange among disciplines.

In recent years, the preservation of ICH has attracted more attention all over the world, followed by a large number of relevant documents where Chinese ICH terms have also been widely used and disseminated. ICH includes folklore, literature, music, dance, drama, handicrafts, medicine, and other categories. There is a large number of cultural proprietary items in ICH terms, such as names of persons, places, articles, art forms, dynasties, classics, as well as widely circulated myths, legends, and historical events. Terminology is the starting point of academic research and theoretical construction. Whether the translation of terms is standardized or not, it directly affects international exchanges (Zeng, 2020). In the context of globalization, the term translation is undoubtedly of great significance for international cultural and academic exchanges and effective communication. The monosemy of terms requires that “a term can only express one concept, and the same concept can only be expressed in one term without a difference” (Feng, 1991). However, the concepts of some terms are vague and misunderstandings and mistranslations occasionally occur. Currently, there are many problems in terminology translation in China, among which the main problem is the lack of semantic textual research and choices (Zhao, 2019). The Chinese translation researcher Gu pointed out that “If (terms) are not translated properly, these terms will destroy the standardization of the original national language, even dominate the host, force some Chinese terms to change their original meaning and succumb to the connotation and extension imposed by foreign terms, and thus causing some strange statements, theories, or adverse atmosphere in Chinese academic circles” (Gu, 1998, p. 6).

In summary, most translation theories and translation researchers have focused their interest on the description and interpretation of translation products (translation products or works), while the research on the production process of translation products (i.e., the psychological process of translation) is rarely seen. As translation theory researchers explained, the essential characteristics of the translation process involve changes that take place in the translator’s psychology in the process of transformation from the source language to the target language; this also involves the relationship between the target language unit and the source language counterpart (Lu et al., 2014). Since ICH terms contain numerous culture-loaded words and expressions, it is feasible to study the English translation of the ICH terms based on metonymy. The main purpose of translating ICH terms is the exchange of culture and to realize the cultural exchange between different countries and nationalities through the translation of terms. Therefore, the translation of ICH terms is related to the development of international cultural and academic exchanges.

## Metonymic mechanism and english translation of chinese intangible cultural heritage terms

### Metonymic mechanism of intangible cultural heritage terms

The purpose of ICH translation is to enable foreign readers to obtain accurate information about the source text to promote the integration of ICH culture with the non-Chinese readers. As a language phenomenon with distinctive regional characteristics and rich historical and cultural connotation, ICH metonymy is an important part of the ICH terminology system as it reflects the unique psychological mode of the Chinese nation and the dynamic formation process of the concept of ICH. However, its profound cultural specificity inevitably causes difficulties for the target language readers to understand. Referring to the translation principles of metaphor in ICH, metonymic translation should also reflect the concept of metonymy and be translated according to its original meaning (Lan, 2010). Therefore, the principles of faithfulness and readability should be taken into account in the English–Chinese translation of ICH terms metonymy. In the English–Chinese translation of ICH terms, the metonymic connotation of the source language should be retained as much as possible, and the vehicle and tenor of the target language should be basically consistent with that of the source text. In the process of the English translation, whether English readers and Chinese readers can produce similar or even the same cognitive experience and cognitive results is an important standard to judge the success of ICH metonymy translation. In sum, two points should be paid attention to regarding the English translation of ICH terms metonymy. The readability of the translation should not be ignored only to emphasize the retention of metonymic characteristics, but also to impose its own cultural system on the target language readers (Zhou, 2019). Bearing in mind that faithfulness does not mean translating terms without any negotiation or alteration. At the same time, to pursue readability, the accuracy of the English translation of ICH terms metonymy should be equally important.

### The embodiment of metonymic mechanism in the English–Chinese translation of intangible cultural heritage terms

Terms are a collection of words used to represent concepts in a specific subject field. ICH terms are the essence of Chinese traditional intangible cultural knowledge. In the process of international communication and exchange of intangible culture, the English translation of Chinese ICH terms is very important, carrying the important mission of foreign publicity and international promotion of Chinese traditional culture. Currently, the research on the English translation of Chinese ICH terms advocates exploring the strategies, principles, and methods of the translation of ICH terms on the premise of preserving the cultural, scientific, national, and historical nature of ICH terms, and trying to find an appropriate balance between the dimensions of faithfulness and smoothness, domestication and foreignization, and seeking common grounds to minimize differences, to realize the standardization of the English–Chinese translation of ICH terms, and promote the internationalization of Chinese ICH.

Translation is also a cross-cultural psychological activity (Yan, 2007). Translation psychology holds that the essence of translation is the psychological process of the translator from cultural conflict to cultural compromise and then to make suitable choices between the two cultures. The cultural compromise is the translator’s understanding and assimilation of the alien culture, and “compromise” is the externalization of the translator’s whole psychological activity.

**FIGURE 1:**
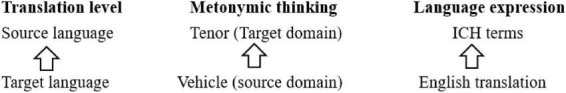
The embodiment of metonymic psychology in Chinese–English translation of ICH terms.

Due to the characteristics of ICH, the difficulties in the translation of ICH terms mainly include two points. First, the asymmetry of language and culture and the cultural metaphor in ICH terms can cause very limited information to be disseminated pertaining to the English-translated texts. ICH texts have the function of cultural load, including specific historical knowledge, typical regional characteristics, and profound cultural connotations, which are the common carriers of cultural values and information resources that are resistant to translation or even untranslatable. For example, 铜器 from the ICH term 艾庄的铜器舞 in China refers to brass percussion instruments in ancient China, such as gongs, cymbals, drums, and clocks; while the English translation of 铜器 is brass instruments referring to brass instruments playing in the Western context (Chen, 2017). The cultural connotation of 舞 from 铜器舞 is also very different from *dance* in the English language. Second, the financial practicability of the ICH English translation takes precedence over the translation of cultural connotation. Taking ICH terms 假发 (Wigs) and 钧瓷 (Jun porcelain) as examples, the English translation of publicity serves for product sales; therefore, their translation focuses on the description of the material, style, price, texture, and quality. The translation of these terms is endowed with a strong sense of universality. Even if there is a small amount of translation of cultural connotation and production skills, it is mainly used to increase the mystery of products and attract foreign customers, and thus improving sales that leads to the financial value of ICH dominating the text translation. That is to say, the contradiction between financial practicability and faithfulness leads to the great weakening of cultural connotation. Therefore, it is urgent to guide and integrate the relevant translation theories to improve the motivation and validity of the English translation of ICH terms and facilitate the communication and dialogue in the cross-cultural communication of ICH. In view of this, this paper, guided by the translation metonymy theory, seeks new ideas for the English translation of ICH terms, especially for some obscure and culturally loaded ICH terms. We believe that metonymy, as an important language conversion mechanism and a psychological cognitive mechanism, has certain applicability in the process of English–Chinese translation of ICH terms (see Figure 1).

According to the definition of the Oxford English dictionary, translation is the transformation from one language to another that can be represented in the transformation between the source language and the target language. For the English translation of ICH terms, the source language is represented as Chinese ICH terms, and the target language is the English translation. According to the cognitive psychological theory, ICH terms in the source language contain a series of possible meanings involved in the translation process, and the translators only select the meaning he/she expects to represent the meaning he/she subjectively understands and then presents it in the form of language expression. Therefore, it can be concluded that metonymic psychology must occur in the process of the transformation of the two languages. The translator places the source language and the target language in the position of Tenor (target domain) and Vehicle (source domain), respectively, and provides psychological channels for cognitive tenor through different vehicles. Different target languages reflect the translator’s different orientations and different metonymic prominence, that is, as two conceptual entities in metonymy operation, one is easy to perceive, understand, and remember compared with the other; the two are unbalanced in cognitive prominence (Wang, 2019, p. 8). Based on the Chinese scholar Xu’s distinction between “understanding one kind of thing in terms of another” and “experiencing one kind of event in terms of another” (Xu, 2018, p. 3), this paper divides the basic factors of metonymic mechanism into “object” and “thing.” The former involves the English translation of ICH nominal terms, and the latter involves the English translation of ICH verbal terms.

### Metonymic mechanism of the English translation of Chinese nominal intangible cultural heritage terms

The nominal terms of ICH mainly express the concept of “thing” (Shi, 2021). According to the theory of translation metonymy, the process of the English translation of ICH nominal terms deals with the proximity between the concept of “thing” in the source language and the target language chosen by the translator through the metonymic mechanism. All cross-language operations based on the mutual generation of the subordinate and the superordinate or the transformation of adjacent relations, such as a part replacing a part belong to the category of translation metonymy (Xu, 2018). The translation metonymy of nominal terms of ICH is mainly realized by replacing a superordinate with a subordinate, a subordinate with a superordinate and a subordinate with a subordinate as discussed further below.

#### Superordinate replaces subordinate

First, some ICH terms in the source language Chinese express their literal meaning more directly, such as “Tiger dance,” “Monkey performance,” “bamboo weaving,” and “Folk paper cutting.” Hence, literal translation can be used to make Chinese corresponds to English expressions. However, because ICH carries specific historical and cultural connotations and has very obvious regionality, some ICH terms are relatively obscure and difficult to understand. Second, it is difficult to accurately convey the connotation of the source language in translation. Metonymic mechanism holds that the superordinate and subordinate are usually expressed in the relationship between superordinate and subordinate categories, and thus using the superordinate to replace the subordinate is to use the superordinate category to refer to the subordinate category.

There are typical examples as 豫剧 (Yuju opera), 跑帷子 (Paoweizi dance), 升旗打酒火 (Shengqi dajiuhuo performance), and 摸摸会 (Momohui customs). Among these ICH terms, opera, dance, performance, and customs are the superordinate categories of *Yuju opera, Paoweizi dance, Shengqi Dajiuhuo performance*, and *momohui customs*, respectively, which shows the relationship between the superordinate and the subordinate. As a superordinate category, “opera, dance, performance, and customs” can refer to all dramas, dances, performances and customs in the world, as well as “Yuju opera,” “Paoweizi dance,” “Shengqi dajiuhuo performance,” and “Momohui customs.”

In translation, using a superordinate category instead of a subordinate category has the following advantages. First, it is conducive to the construction of “Joint Attentional Scenes,” which is easy to be accepted by readers. Second, translation errors can be avoided to a very limited extent by transforming the original text with a higher level of accommodation. However, mistranslation may lead to a greater disaster. If a translator meets “Shenqi Dajiuhuo” and “Momohui” for the first time, he/she does not know how to translate them, but he/she knows that they refer to a kind of performance and custom, respectively, he/she can translate them into the superordinate categories “Performance” and “Customs,” the target language readers can also know the name and category of the source language through translation.

#### Subordinate replaces superordinate

The subordinate replaces the superordinate, which usually refers to the replacement of a subordinate category with a superordinate category. For example, if one does not understand the cultural background of the term 桐蛋制作技艺, the term can be easily misinterpreted as *Paulownia egg making* derived only from the literal meaning, but this translation is very absurd and far from its true meaning. After checking the cultural background of this term, 桐蛋 refers to the duck egg produced in the Tonghe river section of Tonghe town. For this term, the transliteration plus literal translation method in the foreignization translation method should be adopted and translated into “Tongdan duck egg making,” where “egg” is an upper category and “duck egg” is a lower category. The former and the latter constitute a superordinate and a subordinate relationship, respectively. Using the subordinate category “duck egg” to refer to the superordinate category “egg” is a translation metonymy phenomenon of substituting a subordinate for the superordinate. It can be seen that one of the important reasons why the translation process is metonymic because the source language itself involves the use of metonymic thinking. Therefore, translators have to adapt to the thinking path of the source language and use metonymic thinking to solve translation problems in the process of translation.

#### Subordinate replaces subordinate

Traditional transliteration belongs to metonymic translation based on phonetic proximity, that is, the target language is transferred to the source language through the phonetic form. Phonetic metonymy translation is common in the English translation of Chinese ICH terms, and transliteration is applicable to some Chinese words that have been named in the early development process and are well known to many foreigners and are even included in foreign dictionaries (Bian and Du, 2019), such as 少林功夫 *(Shaolin Kungfu)*, 太极拳 *(Taijiquan)*, and 唐三彩 *(Tang Sancai)*. The corresponding expression of some Chinese ICH terms cannot be found in English because ICH originated from Chinese history and traditional culture, for instance, ICH terms include 玛纳斯 *(Manas)*, 花儿 *(Hua’er)*, 古琴 *(Guqin)*, 南音 *(Nanyin)*, and 麦西热普 *(Meshrep)*. These transliterated words were included in the book *Intangible Cultural Heritage in China*, published by Beijing Language and Culture University Press in 201*7* (China Intangible Cultural Heritage Project, 2017). ICH terms belong to a heterogeneous culture, and it is difficult to find the corresponding language expression in the English language. Therefore, metonymy based on phonetic proximity has become the preferred strategy for translating such terms by highlighting the unique ICH culture.

### Metonymic mechanism of the English translation of Chinese intangible cultural heritage verbal terms

The ICH verbal terms highlight the “event” and mainly express the concept of “event” (Shi, 2021). According to the theory of translation metonymy, the English translation of ICH verbal terms is to regard the whole event as a process and realize the purpose of the English translation by highlighting some aspects of the process through the metonymic mechanism. The English translation of the ICH term “正骨疗法” is considered here as an example (see Table 2). According to the metonymic types, the target language is summarized herein. The ICH cases cited in this paper are from the website of the China Guangdong Culture Museum, and the translation of the term “正骨” is from the website (termonline.cn). Through the analysis, we can draw the following conclusions: (1) At present, there is no unified standard for the English translation of 正骨疗法, and there are two versions; (2) Different versions reflect different types of metonymy; (3) Different translations reflect different metonymic prominence. The Chinese ICH term 正骨疗法 comes from the list of ICH in Guangdong, China, and it is defined by the official website of the National Science and Technology Terminology Examination and Approval Committee as “various medical methods for the treatment of muscle and bone injuries such as fracture and dislocation.” According to the metonymic view of translation, the event of 正骨疗法 is regarded as a process, then the two translations in Table 2 reflect the metonymic translation of some generations as a whole, but they have different highlights. The first translation in Table 2 refers to the process in the way of treatment to highlight the role of treatment. The second translation refers to the process in the way of function that highlights the function of “Bone adjusting” due to the use of the noun structure of *Bone adjusting*. In the process of the English translation, it does not only reflect the metonymic mechanism of partial substitution for the whole within the event category, but it also reflects a subordinate to a superordinate or a subordinate to a subordinate substitution in the English translation of nominal ICH terms. Another example is the Guangdong ICH term 粤语说古 as the metonymic verbal with which there are multiple vehicles to form a metonymic relationship, resulting in multiple translations as listed below:

**TABLE 2:** English translation analysis of ICH “正骨疗法.”

Target language	Metonymic type
(1) Therapy of adjusting bone	Manner replaces process
(2) Therapy of bone-adjusting	Function replaces process

Translation 1: The customs of telling ancient stories in Cantonese.
Translation 2: Sharing ancient stories in Cantonese.

The character 说 from the term 粤语说古 reflects the connection between *Cantonese* and *stories*. This connection can be interpreted as different event processes, such as “tell,” “share,” and “act out.” Different interpretations are reflected through different translations, such as tell, share, and act out, which reflect the metonymic way of the substitution process. For example, translation number 1 highlights the role of conveying stories in Cantonese; translation number 2 highlights the process of sharing stories in Cantonese. The first translation replaces “telling stories” with “the custom of telling stories,” which belongs to the metonymy of a subordinate replacing a superordinate. From the first and second translations, 古 from the ICH term 说古 has been translated into *ancient stories*, which belongs to the metonymic translation of replacing a subordinate with a superordinate, highlighting the connotative characteristics of 古.

The ICH term 摇快船 is another example; the Chinese character 摇 can be translated as “row” which is a verb; therefore, it seems that 摇快船 can be translated into *Row fast boat* literally. However, according to the cultural background of the term “摇快船,” it was formed at the beginning of the Qing dynasty, which is the custom in the Qingpu area of Shanghai city during the temple fair on the 15th day of the 7th lunar month. Therefore, the term 摇快船, in essence, is a kind of boat racing. Accordingly, the term 摇快船 has been translated into *Boat racing* which is listed in the *Album of the list of Shanghai Intangible Cultural Heritage Items* published by a publishing house of Shanghai municipal administration of culture, radio, film, and television in 2010 (Picture Album of Shanghai Municipal Administration of Culture, 2010). According to the metonymic view of translation, the event 摇快船 is regarded as a process, so the translation of “Boat racing” reflects the metonymic translation of replacing a superordinate with a subordinate by using the nominal structure “Boat racing” to transfer the process to a purpose, and highlight the role of 摇.

## Discussion and conclusion

The translation of ICH terms based on cognitive psychology defines the metonymic mechanism between the target language and the source language counterpart as a translational relationship. To be precise, the relationship between the target language unit and the source language counterpart is a metonymic process, or metonymy is the internal psychological motivation and cognitive mechanism of the transformation from the source language to the target language to bring the relationship between the target language and the source language into a unified interpretation framework in the process of translation.

This paper attempts to organically combine the metonymic cognitive process with the English translation of ICH terms and explore the psychological processes, methods, and strategies of the English translation of ICH terms to provide a new English translation scheme for the polysemy and diversity in the process of interpretation of ICH terms. According to the metonymic mechanism, the proximity relationship between the source ICH terms and the corresponding items of the English target language triggers metonymic psychology. This metonymic mechanism can be used to construct the cross-language transformation relationship to provide translators with a translatable psychological processes. This paper finds that the English translation of ICH nominal terms, to some extent, is based on the metonymy of replacing a superordinate with a subordinate, a subordinate with a superordinate, or a subordinate with a subordinate. The English translation of ICH verbal terms mainly regards the whole verbal action event as a process, which can be realized through the metonymic mechanism of replacing a superordinate with a subordinate within the event category. The metonymic mechanism has practical guiding significance for the English translation practice of Chinese ICH terms.

As a new paradigm of translation research, cognitive translatology uses the theories and methods of cognitive psychology, cognitive linguistics, and other branches of cognitive science to explain translation phenomena to reveal the cognitive process of translation, which is deemed as the essence of translation (Lu and Wang, 2013). The study of translational metonymy in this paper is a useful attempt to explain the relationship between the corresponding items of the target language and the source language in the process of psychological activities of translation by using the metonymic mechanism of cognitive linguistics. Specifically, it uses the metonymic psychological mechanism to explain the internal psychological motivation and cognitive mechanism behind translation skills such as substitution.

The main contributions of this paper are as follows: first, this paper holds that the essence of the translation process of ICH terms is psychological rather than material, explored the metonymic mechanism of ICH terms, and offered some references for culture-loaded text translation, which further promoted the scope of relevant research by Dan (2016); Qiu and Feng (2019), and Ren (2019). Second, the translation process of ICH terms based on cognitive psychology mainly includes the translator’s understanding of the source language, the choice of vocabulary, the coding of knowledge, and the revision of the translation that are practical and meaningful for the translators to enhance their translation cognitive competence, and improve their understanding of the translation process that made a contribution to the existing literature by Ulvydiene (2013); Man (2021), and Zhou (2021). Only by understanding the translation process, the translator can improve his/her translation skills and the level of translation.

The conclusion of this paper is as follows: First, in the process of translation, there is a metonymic relationship between the target language of ICH terms and the corresponding items of the source language; that is, there is a semantic relationship between them; second, metonymic mechanism holds that metonymy is an important psychological motivation and cognitive mechanism behind translation skills such as substitution which brings the relationship between the corresponding items of the target language and the source language into a unified interpretation framework, and this, in turn, has certain guiding significance for the research of translation theory and translation practice.

## Implications and limitations

The findings of this research can be utilized by various stakeholders, including teachers of translation courses, learners, and the administrators of the ICH field. These findings have various implications for the translation of ICH terms, as well as for further research on this topic. First, this study reveals that it is feasible to improve the Chinese–English translation of the ICH terms from the perspective of metonymy; second, it is also practical to improve the translational skills of ICH terms based on metonymic mechanism, as long as attention is needed on the methodology to cultivate translators’ translative competency; third, these findings would assist the relevant efforts in the promotion of ICH of going abroad.

The results of this study support its research objectives. However, there are still some limitations regarding its research conditions and methods as we only included Chinese ICH terms as our samples. Therefore, future research should explore other countries’ ICH terms to expand the scope of these findings. Furthermore, based on our findings, the next research step would be to explore the teaching efficiency of applying metonymic mechanism to the translation of ICH based on qualitative methods to put forward specific and practical models to improve the quality and feasibility of the integration of ICH translation and metonymy.

## Data availability statement

The original contributions presented in this study are included in the article/supplementary material, further inquiries can be directed to the corresponding author.

## Author contributions

LJ conceived the manuscript’s framework and structure and also wrote up the main body of the manuscript. GA-S provided feedback and did language and formatting editing. FY, MC, and MH did proofreading of the manuscript. All authors contributed to the article and approved the submitted version.

## Conflict of interest

The authors declare that the research was conducted in the absence of any commercial or financial relationships that could be construed as a potential conflict of interest.

## Publisher’s note

All claims expressed in this article are solely those of the authors and do not necessarily represent those of their affiliated organizations, or those of the publisher, the editors and the reviewers. Any product that may be evaluated in this article, or claim that may be made by its manufacturer, is not guaranteed or endorsed by the publisher.
